# GDF15 Modulates the Zoledronic-Acid-Induced Hyperinflammatory Mechanoresponse of Periodontal Ligament Fibroblasts

**DOI:** 10.3390/cells13020147

**Published:** 2024-01-12

**Authors:** Ann Nitzsche, Christoph-Ludwig Hennig, Katrin von Brandenstein, Annika Döding, Ulrike Schulze-Späte, Judit Symmank, Collin Jacobs

**Affiliations:** 1 Department of Orthodontics, University Hospital Jena, Leutragraben 3, 07743 Jena, Germany; ann.nitzsche@med.uni-jena.de (A.N.); christoph-ludwig.hennig@med.uni-jena.de (C.-L.H.); katrin.brandenstein@med.uni-jena.de (K.v.B.); collin.jacobs@med.uni-jena.de (C.J.); 2 Section of Geriodontics, Department of Conservative Dentistry and Periodontics, University Hospital Jena, Leutragraben 3, 07743 Jena, Germany; annika.doeding@med.uni-jena.de (A.D.); ulrike.schulze-spaete@med.uni-jena.de (U.S.-S.)

**Keywords:** zoledronic acid, orthodontic tooth movement, periodontal ligament fibroblasts, GDF15, senescence, DNA damage response, inflammatory mechanoresponse

## Abstract

Orthodontic tooth movement (OTM) is thought to be impeded by bisphosphonate (BP) therapy, mainly due to increased osteoclast apoptosis and changes in the periodontal ligament (PdL), a connecting tissue between the alveolar bone and teeth. PdL cells, mainly fibroblasts (PdLFs), are crucial regulators in OTM by modulating force-induced local inflammatory processes. Recently, we identified the TGF-β/BMP superfamily member GDF15 as an important modulator in OTM, promoting the pro-inflammatory mechanoresponses of PdLFs. The precise impact of the highly potent BP zoledronate (ZOL) on the mechanofunctionality of PdLFs is still under-investigated. Therefore, the aim of this study was to further characterize the ZOL-induced changes in the initial inflammatory mechanoresponse of human PdLFs (hPdLFs) and to further clarify a potential interrelationship with GDF15 signaling. Thus, two-day in vitro treatment with 0.5 µM, 5 µM and 50 µM of ZOL altered the cellular properties of hPdLFs partially in a concentration-dependent manner. In particular, exposure to ZOL decreased their metabolic activity, the proliferation rate, detected using Ki-67 immunofluorescent staining, and survival, analyzed using trypan blue. An increasing occurrence of DNA strand breaks was observed using TUNEL and an activated DNA damage response was demonstrated using H2A.X (phosphoS139) staining. While the osteogenic differentiation of hPdLFs was unaffected by ZOL, increased cellular senescence was observed using enhanced p21^Waf1/Cip1/Sdi1^ and β-galactosidase staining. In addition, cytokine-encoding genes such as *IL6*, *IL8*, *COX2* and *GDF15*, which are associated with a senescence-associated secretory phenotype, were up-regulated by ZOL. Subsequently, this change in the hPdLF phenotype promoted a hyperinflammatory response to applied compressive forces with an increased expression of the pro-inflammatory markers *IL1β*, *IL6* and *GDF15*, as well as the activation of monocytic THP1 cells. GDF15 appeared to be particularly relevant to these changes, as siRNA-mediated down-regulation balanced these hyperinflammatory responses by reducing IL-1β and IL-6 expression (IL1B *p*-value < 0.0001; IL6 *p*-value < 0.001) and secretion (IL-1β *p*-value < 0.05; IL-6 *p*-value < 0.001), as well as immune cell activation (*p*-value < 0.0001). In addition, ZOL-related reduced RANKL/OPG values and inhibited osteoclast activation were enhanced in *GDF15*-deficient hPdLFs (both *p*-values < 0.0001; all statistical tests: one-way ANOVA, Tukey’s post hoc test). Thus, GDF15 may become a promising new target in the personalized orthodontic treatment of bisphosphonatepatients.

## 1. Introduction

During the functional physiological and pathological transformation of the jaws, alveolar bone remodeling occurs depending on the forces applied to the bone and the periodontal ligament (PdL). Therefore, regulated bone formation and bone resorption is mutually dependent on the activation of osteoblasts and osteoclasts and the adaptation of the PdL [1,2]. In general, the PdL is a connective tissue between the alveolar bone and the teeth consisting of a large diverse population of cells, including fibroblasts as the main cell type [3]. These PdL fibroblasts (PdLFs) have many osteoblast-like properties, such as the expression of the osteogenic markers alkaline phosphatase (ALP) and runt-related transcription factor 2 (RUNX2) and the formation of mineral-like nodules in vitro [4,5,6].

During orthodontic tooth movement (OTM), the remodeling of the PdL and physiological adaptation of the alveolar bone are prompted by applying mechanical forces via orthodontic appliances [1,7]. These forces trigger the mechanobiological responses of the PdL cells, which promote a local aseptic and transient inflammatory microenvironment that promotes tissue and bone remodeling [1,8]. Therefore, compressive forces typically promote bone-resorbing conditions by inducing hypoxia and activating the pro-inflammatory response of the PdLFs, including an increase in cytokines such as interleukin 1β (IL-1β), IL6, IL8 and cyclooxygenase 2 (COX2) [9,10,11]. In this process, increased osteoclasts are also recruited from the adjacent bone marrow spaces and activated by specific signals, leading to increased local degradation of the alveolar bone [12]. In this regard, the RANK/RANKL/OPG system is an important pathway, with PdLFs secreting increasing levels of receptor activator of NF-κB ligand (RANKL), stimulating osteoclast differentiation [13,14].

Recent studies by our group and Li et al. have reported that also growth/differentiation factor 15 (GDF15) plays a major role in OTM [15,16,17]. GDF15 is a member of the TGF-β/BMP superfamily and is released by various cell types and tissues during metabolic, mechanical or chemical stress [18,19,20,21,22]. Consistently, GDF15 is also up-regulated by the mechanical stimulation of human periodontal ligament fibroblasts (hPdLFs), and promotes their pro-inflammatory response [2,16,17,23]. In addition to modulating osteogenic cell differentiation and inflammation in the PdL, GDF15 is also involved in the regulation of cell death and cellular senescence, as well as aging [2,15,16,19,24,25].

OTM can be interfered by a number of pharmaceuticals, which may also induce functional disturbances that affect bone resorption or attachment processes [26,27]. One of these pharmaceuticals is the bisphosphonate zoledronate (ZOL), which is mainly used in the treatment of cancerous bone metastases, bone metabolic diseases, as well as in osteoporosis therapy [28,29,30]. Bisphosphonates are internalized in osteoclasts, leading to their apoptosis. This affects the bone metabolism with possible side effects such as antiresorptive-agent-related osteonecrosis of the jaw [31,32,33,34]. As the orthodontic patient population experiences a demographic shift toward higher ages, bisphosphonate interactions are increasingly becoming a major focus within orthodontic therapy [35,36]. The effects of ZOL on cell viability and proliferative capacity are well described for several cell types, particularly for osteoclasts [37,38]. Its influences on DNA damage, the DNA damage response and the induction of senescence have also been investigated in several cell types [39,40]. Furthermore, animal experiments have already shown effects such as reduced tooth movement, fewer osteoclasts, widened periodontal ligaments and high bone mineralization under bisphosphonates during OTM [36,41,42,43,44,45]. In many previous studies, OTM is thought to be reduced by BP therapy, mainly due to the lower inflammatory-induced resilience of the periodontium, the reduction in osteoclasts, cytoplasmic polarity and morphological changes in the PdL [43,45,46,47,48]. However, the precise impact and effects of ZOL during OTM on the mechanorelated functions of PdL fibroblasts have been inadequately investigated. Therefore, the aim of this study was to characterize the effects of ZOL on the inflammatory mechanoreaction modulated by human PdL fibroblasts and further determine the potential effects of GDF15.

## 2. Materials and Methods

### 2.1. Cell Culture

The commercially acquired human periodontal ligament fibroblasts (hPdLF, Lonza, Basel, Switzerland) are a pooled cell line from several adult donors of Eurasian origin and were used in this study from passage three to five. They were cultured in DMEM high glucose (4.5 g/L) with stable glutamine (DMEM-HPSTA, Capricorn Scientific, Ebsdorfergrund, Germany) and supplemented with 10% heat-inactivated fetal bovine serum (FBS, Thermo Fisher Scientific, Carlsbad, CA, USA), penicillin (100 U/mL)/streptomycin (100 U/mL) and L-ascorbic acid (50 mg/L) at cell growth conditions (37 °C, 5% CO_2_, 95% humidity). When a confluency of 75% was reached, the hPdLFs were detached with 0.05% Trypsin/EDTA (Thermo Fisher Scientific, Carlsbad, CA, USA), and reseeded for further growth or experimental use (48-well plates: 2.5 × 10^3^ cells per well on glass coverslips, 6-well plates: 10^4^ cells per well). Experimental stimulation was performed when the cells reached 75% confluency. To culture the THP1 monocytic cells (DSMZ, Braunschweig, Germany) at 37 °C, 5% CO_2_ and 95% humidity, RPMI 1640 medium (RPMI, Capricorn Scientific, Ebsdorfergrund, Germany) supplemented with 10% FBS and penicillin (100 U/mL)/streptomycin (100 U/mL) was used. Each week, the non-adherent cells were subdivided and reseeded at a density of 1 × 10^6^ cells in 20 mL of medium in a T175 culture flask (Thermo Fisher Scientific, Carlsbad, CA, USA).

### 2.2. Stimulation with Zoledronic Acid (ZOL)

For subsequent analysis regarding to the impact of zoledronate, the hPdLFs were treated with 50 µM, 5 µm and 0.5 µM zoledronic acid monohydrate (ZOL; Merck Millipore, Burlington, MA, USA) for 48 h prior to fixation or compressive force application. The ZOL was dissolved in H_2_O, which was also used for the respective controls.

### 2.3. MTT Assay

A MTT (3-(4,5-dimethylthiazol-2-yl)-2,5-diphenyl tetrazolium bromide) colorimetric assay (Merck Millipore, Burlington, MA, USA) was applied according to the manufacturer’s guidelines to determine the metabolic activity. Thus, the optical density was analyzed using the Infinite M Nano (Tecan Austria GmbH, Gröding, Austria).

### 2.4. Trypan Blue Staining

To analyze the cell viability, trypan blue staining was performed. Following a washing step with pre-warmed Phosphate-Buffered Saline (PBS), the unfixed hPdLFs were cultured for 5 min with 1:2 trypan blue (Trypan Blue Stain 0.4%, Thermo Fisher Scientific, Carlsbad, CA, USA) in DMEM, directly followed by microscopic imaging.

### 2.5. Immunofluorescent Staining

Immunofluorescent staining was performed as previously described [49,50,51]. Briefly, following 10 min fixation with paraformaldehyde (PFA, 4%), three washing steps in 1× PBS/0.1% Triton X (Triton X^®^ 100, Carl Roth GmbH + Co. KG, Karlsruhe, Germany) were applied. To block the unspecific antibody-binding sites, 4% bovine serum albumin (BSA, SEQENS, H2B/ESTER Technopole, Cedex, Limoges, France) in 1× PBS/0.1% Triton X was used. Subsequently, primary antibody incubation was performed for 3 h in blocking buffer (4% BSA/1× PBS/0.1% Triton X), followed by washing in 1× PBS/0.1% Triton X and secondary antibody incubation for 45 min in blocking buffer. After another PBS washing step, the cell nuclei were stained with DAPI (1:10,000 in PBS), followed by rinsing with PBS and water. Finally, coverslips were embedded with Mowiol^®^4-88 onto glass object slides for microscopic imaging. The following primary antibodies were used: rabbit anti-Ki67 (1:250, Abcam, Cambridge, UK), rabbit anti-p21^Waf1/Cip1/Sdi1^ (Cell Signaling Technology, Leiden, The Netherlands), H2A.X (phosphoS139) and rabbit anti-GDF15 (both Thermo Fisher Scientific, Carlsbad, CA, USA). As secondary antibodies, goat anti-rabbit Alexa Fluor 488 and goat anti-mouse Cy5 (1:1000: Jackson ImmunoResearch, West Grove, PA, USA) were used. Alexa Fluor 594 Phalloidin (Thermo Fisher Scientific, Carlsbad, CA, USA) was used to label the F-actin.

### 2.6. RNA Extraction and cDNA Synthesis

After cell isolation using TRIzol (Thermo Fisher Scientific, Carlsbad, CA, USA), RNA extraction was performed as previously described [52]. To this end, the RNA Clean & Concentrator-5 kit (Zymo Research, Freiburg, Germany) was applied according to the manufacturer’s guidelines. The RNA quality and quantity were assessed using the NanoDrop One© (Thermo Fisher Scientific, Carlsbad, CA, USA). The SuperScript IV Reverse Transcriptase and Oligo (dt)18 primers (both Thermo Fisher Scientific, Carlsbad, CA, USA) were used to synthetize the cDNA according to the manufacturer’s protocol.

### 2.7. Quantitative Polymerase Chain Reaction (PCR)

For the expression analysis, we used Luminaris Color HiGreen qPCR Master Mix (Thermo Fisher Scientific, Carlsbad, CA, USA). The primer design and validation were performed as previously described [2,15]. All the primers (Eurofins Genomics, Ebersberg, Germany) we used in our study are listed in Table 1, generating products between 120 and 190 bp in length. As reference genes, we used *RPL22* and *TBP*. The quantitative PCR was performed using the qTOWER^3^ (Analytik Jena, Jena, Germany) according to the manufacturer’s guidelines. For the analysis, the efficiency-corrected ∆∆CT method was used [53].

### 2.8. Alkaline Phospatase Activity Analysis

For the determination of osteogenic differentiation, an analysis of the alkaline phosphatase activity was performed using staining with 1-Step™ NBT/BCIP Substrate Solution (Thermo Fisher Scientific, Carlsbad, CA, USA) for 90 min. The cells were directly imaged after staining.

### 2.9. TUNEL Assay

The ApopTag^®^ Fluorescein In Situ Apoptosis Detection Kit (Merck Millipore, Burlington, MA, USA) was used according to the manufacturer’s protocol to specifically label cells with DNA strand breaks associated with subsequent apoptosis.

### 2.10. β-Galactosidase Staining

To detect the cellular senescence, the hPdLFs were fixed in 4% PFA for 10 min and stained using the CellEvent™ Senescence Green Detection Kit (Thermo Fisher Scientific, Carlsbad, CA, USA) according to the manufacturer’s protocol.

### 2.11. Mechanical Compression

For the RNA isolation, a compressive force of 2 g/cm^2^ was applied for 24 h to the cells grown in 6-well plates in culture conditions as previously performed [15]. For all the other analytical experiments performed in the 48-well plates, compression was performed with the minimum technically possible force of 7.13 g/cm^2^ using centrifugation (5810 R, Eppendorf, Hamburg, Germany) at 30 °C for 24 h, with a 3 h break at culture conditions after 12 h centrifugation. Likewise, the control cells were cultured at 30 °C for each 12 h interval.

### 2.12. siRNA-Mediated Knockdown of GDF15

Lipofectamine^TM^ 2000 (Thermo Fisher Scientific, Carlsbad, CA, USA) was used for the transfection of siRNA as previously described [15]. Briefly, siRNA oligos targeting human *GDF15* (50 nM, Santa Cruz Biotechnology, Dallas, TX, USA) were added to the living cells for five hours in an Opti-MEM I-reduced serum medium (Thermo Fisher Scientific, Carlsbad, CA, USA) containing 100 U/mL penicillin and 100 µg/mL streptomycin at culture conditions. For the control siRNA, 50 nM of BLOCK-iT Alexa Fluor green control siRNA (Thermo Fisher Scientific, Carlsbad, CA, USA) was used, targeting no human transcript. Prior to further treatment, the standard culture medium was applied.

### 2.13. THP1 Activation Assay

To visualize the cytokine secretion of the stimulated hPdLFs, activation of the monocytic THP1 cells was performed like previously described [16,49,50,52]. Briefly, Celltracker CMFDA (15 µM, 30 min, Thermo Fisher Scientific, Carlsbad, CA, USA)-stained non-adherent THP1 cells were applied for 30 min to the hPdLFs cultured on the coverslips. Washing with pre-warmed PBS (Thermo Fisher Scientific, Carlsbad, CA, USA) removed any non-attached THP1 cells. The coverslips were fixed in 4% PFA for 10 min prior to PBS washing and nuclei staining (DAPI, 1:10,000 in PBS, Thermo Fisher Scientific, Carlsbad, CA, USA) Finally, the coverslips were embedded with Mowiol (Carl Roth, Karlsruhe, Germany) for the microscopic analysis.

### 2.14. Osteoclast Activation Assay and Tartrate-Resistant Acid Phosphatase Staining

TRAP staining was performed to analyze the hPdLF-modulated osteoclast activation as previously described [16]. Briefly, the THP1 cells were pre-stimulated with phorbol 12-myristate 13-acetate (PMA, 100 ng/mL) for macrophage differentiation prior to exposure to the supernatant of stimulated hPdLF medium for six days. The medium supernatants were applied 1:1 with fresh THP1 culture medium. After fixation with 4% PFA for 10 min and 50:50 acetone/ethanol for 1 min and air-drying, staining for tartrate-resistant acid phosphatase (TRAP) with 0.1 mg/mL Naphtol AS-MX phosphate, 0.5 mg/mL Fast Red Violet LB salt, 1% N,N-dimethyl formamide in 50 mM sodium acetate trihydrate, 50 mM tartrate dehydrate and 0.1% acetic acid (all Merck Millipore, Burlington, MA, USA) was performed for 60 min at 37 °C. Subsequently, microscopic imaging was performed.

### 2.15. Enzyme-Linked Immunosorbent Assay (ELISA)

For the characterization of the secreted cytokine levels, the medium supernatants of the treated hPdLFs were analyzed using specific ELISAs for GDF15, IL-1β, IL-6, OPG and RANKL (all Abcam, Cambridge, UK) according to the manufacturer´s guidelines.

### 2.16. Microscopy and Image Analysis

All the coverslips were scanned using the laser scanning microscope TCS SP5 (Leica, Wetzlar, Germany). The Fiji Image J software (https://imagej.net/software/fiji; accessed on 10 January 2021, version number 1.52p) was used for the image analysis. The intensity measurements of p21^Waf1/Cip1/Sdi1^, H2A.X and GDF15 were performed as previously described [49,50]. Briefly, to avoid influencing the respective expression intensities, individual experiments were microscopically scanned on the same day using pre-warmed lasers and identical settings for each treatment condition. The most intense condition was determined in each experiment prior to scanning and used as calibration to avoid overexposure. The mean grey values (MGVs) of immunofluorescent staining were measured in 90 cells in total for each condition. For the correction of each cell intensity, the background was measured and subtracted from their MGVs. The intensities of the MGVs were visualized as thermal LUTs. Each MGV was normalized to the mean MGV of the respective control condition for each biological replicate and presented as a percent change in the images. The figure design was conducted using Adobe Photoshop CS5 (https://adobe.com; accessed on 10 October 2021).

### 2.17. Statistics

Each in vitro experiment was performed using at least biological triplicates, characterized by different subcultures of cells, with technical duplicates for each condition in each independent experiment. Graph Pad Prism 9 (https://www.graphpad.com; accessed on 10 October 2021, version number: 10.1.2) was used for the statistical analysis. As statistical tests, Student´s *t*-test or one-way ANOVA with a post hoc test (Tukey) was applied as indicated in the specific figure legends. Significance levels: * *p* < 0.05; ** *p* < 0.01; *** *p* < 0.001.

## 3. Results

### 3.1. Zoledronic Acid Affects the Viability and Proliferation of hPdLFs but Not Their Differentiation

Based on the previously published substantial influences of zoledronic acid (ZOL) on various cell types with respect to their cellular characteristics [39,45,54,55,56,57,58,59], we first performed the appropriate analyses in hPdLFs to evaluate their response to 48 h of ZOL exposure. To determine the effects of ZOL, increasing concentrations (0.5 µM, 5 µM, 50 µM) were used for the stimulation of the cultured fibroblasts.

**Figure 1:**
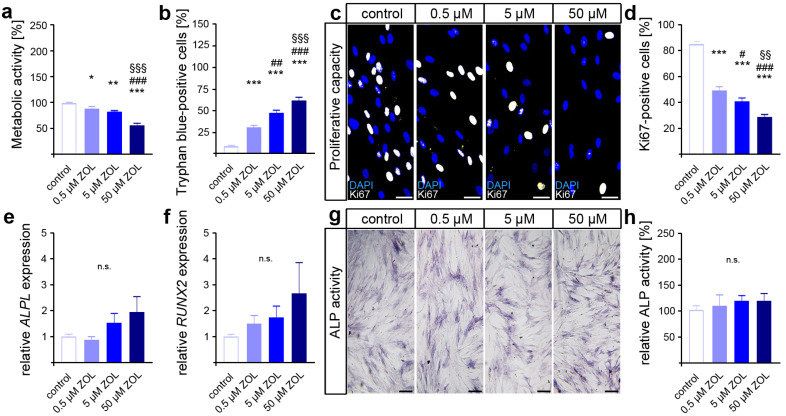
Zoledronic acid limits cell viability and proliferation of human PdL fibroblasts. In vitro-cultured hPdLFs were exposed to zoledronic acid (ZOL) at increasing concentrations of 0.5 µM, 5 µM and 50 µM for two days. (**a**) ZOL-induced reduced metabolic activity. Data are displayed in relation to the control. (**b**) ZOL increased the number of trypan-blue-positive cells. (**c**,**d**) ZOL reduced the number of Ki67-positive (white) proliferative hPdLFs. Nuclei are stained with DAPI (blue). (**e**,**f**) Quantitative analysis revealed no ZOL-induced impact on *ALPL* and *RUNX2* expression levels in stimulated hPdLFs encoding osteogenic markers. (**g**,**h**) ALP activity analysis revealed no influence of ZOL treatment in stimulated hPdLFs. Data are displayed in relation to the control. */# *p* < 0.05; **/##/§§ *p* < 0.01; ***/###/§§§ *p* < 0.001; */**/*** in relation to control, #/##/### in relation to 0.5 µM ZOL, §§/§§§ in relation to 5 µM ZOL; n.s., not significant. One-way ANOVA with post hoc test (Tukey’s). Scale bars: 25 µm.

As an indicator for cell viability and proliferation, as well as compound cytotoxicity [60,61], the metabolic activity was first analyzed via MTT assay. Compared to the control, reduced levels of metabolic activity were detected in the two-day ZOL-treated hPdLFs (Figure 1a). Therefore, the highest ZOL concentration resulted in the lowest metabolic activity. To further analyze the cell viability, we performed trypan blue staining on the ZOL-treated hPdLFs (Figure 1b). Higher numbers of trypan-blue-positive cells were observed with increasing ZOL concentrations, suggesting the cytotoxic effect of ZOL.

Next, we investigated the impact of ZOL on the proliferative capacity of hPdLFs using immunofluorescence staining of the proliferation marker Ki67 (Figure 1c,d). Thereby, a decreasing number of proliferative fibroblasts were detected with an increasing ZOL concentration, pointing to the limiting effect of ZOL on the cell proliferation of hPdLFs.

Since specific stimuli trigger the differentiation of PdL fibroblasts into osteoblasts, we investigated the effects of ZOL in regard to their cell fate. To this end, quantitative expression analysis of the genes encoding the osteogenic differentiation marker alkaline phosphatase (ALP, gene: *ALPL*) and runt-related transcription factor 2 (RUNX2) was performed (Figure 1e,f). However, no significant differences in gene transcription were detected after 48 h of ZOL treatment. Additionally, we analyzed the activity of ALP as a marker for active osteoblasts (Figure 1g,h). Consistent with the expression levels of osteogenic markers, no significant changes in calcium deposition were observed after ZOL exposure either. Taken together, these data suggest that zoledronic acid dose-dependently limits the viability and proliferation of PdL fibroblasts without affecting the osteogenic differentiation of those cells.

### 3.2. Zoledronate Activate DNA Damage Response and Promote Cellular Senescence in hPdLFs

Given the conflicting results on the effect of zoledronate on the DNA damage response (DDR) in different cell types and the relevant role of this stress response in PdL health and functionality [62,63,64], we next focused on DDR activation in ZOL-treated hPdLFs.

**Figure 2:**
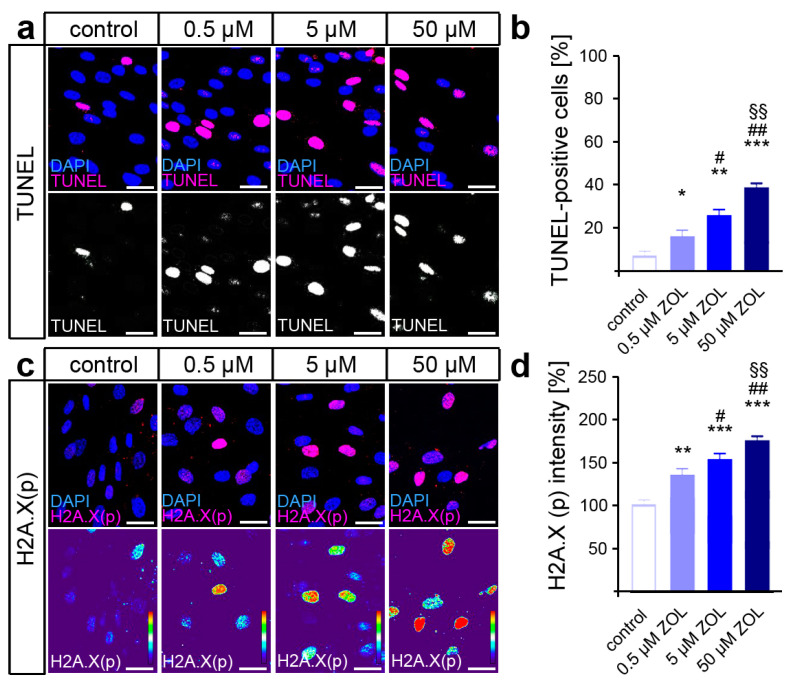
ZOL induces DNA strand breaks and DNA damage response. (**a**,**b**) Two-day treatment of hPdLFs with increasing concentrations of zoledronic acid (ZOL) enhanced the number of TUNEL-positive cells (magenta upper panel, white lower panel) indicating DNA strand breaks. Nuclei are stained with DAPI (blue). (**c**,**d**) ZOL increased the intensity of H2A.X (phosphoS139) (magenta upper panel, intensity staining lower panel), pointing to an activated DNA damage response. Data are displayed in relation to the control. */# *p* < 0.05; **/##/§§ *p* < 0.01; *** *p* < 0.001; */**/*** in relation to control, #/## in relation to 0.5 µM ZOL, §§ in relation to 5 µM ZOL. One-way ANOVA with post hoc test (Tukey’s). Scale bars: 25 µm.

Terminal deoxynucleotidyl transferase dUTP nick end labeling (TUNEL) was performed to detect the DNA strand breaks caused by ZOL treatment, revealing a dose-dependent increase in DNA damage (Figure 2a,b). Since the phosphorylation of H2A.X by DDR kinases is considered a reliable marker of active DDR [65], immunofluorescence staining of H2A.X (phosphoS139) was performed subsequently (Figure 2c,d). Thus, ZOL caused an increased phosphorylation rate of H2A.X in the hPdLFs in a dose-dependent manner, indeed suggesting enhanced DDR activation in the hPdLFs.

**Figure 3:**
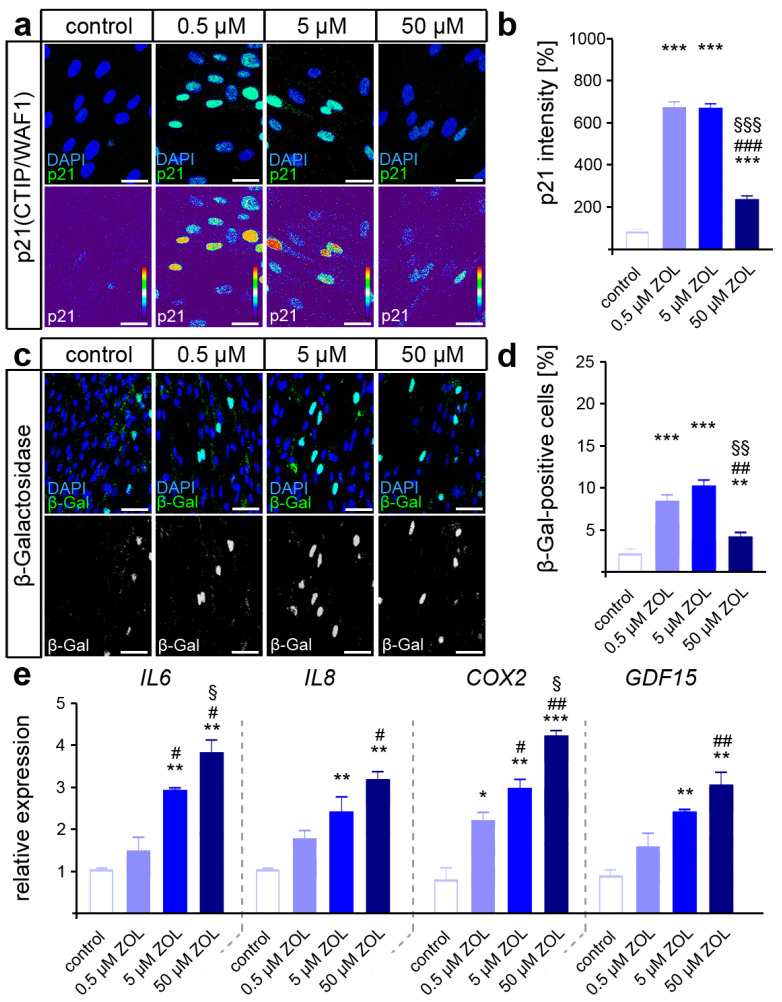
ZOL treatment promotes cellular senescence of hPdLFs. (**a**,**b**) Two-day treatment of hPdLFs with increasing concentrations of zoledronic acid (ZOL) enhanced the intensity of p21^(CTIP/WAF1)^ (green upper panel, intensity staining lower panel), analyzed in relation to the control. Nuclei are stained with DAPI (blue). (**c**,**d**) ZOL increased the number of β-galactosidase (β-Gal)-positive cells (green in upper panel, white in lower panel), indicating up-regulated cellular senescence. (**e**) Quantitative analysis revealed increased expression levels of the genes *IL6*, *IL8*, *COX2* and *GDF15* encoding pro-inflammatory cytokines. */#/§ *p* < 0.05; **/##/§§ *p* < 0.01; ***/###/§§§ *p* < 0.001; */**/*** in relation to control, #/##/### in relation to 0.5 µM ZOL, §/§§/§§§ in relation to 5 µM ZOL. One-way ANOVA with post hoc test (Tukey’s). Scale bars: 25 µm.

Persistently, the increased activation of DDR signaling may trigger cellular senescence, which may compromise the functionality of hPdLFs [39,66]. To test this, immuno-fluorescent staining of the senescence-associated cyclin-dependent kinase (CDK) inhibitor p21^Waf1/Cip1/Sdi1^ was performed. Indeed, a dose-dependent increase in p21 intensity was detected in the hPdLFs due to ZOL exposure (Figure 3a,b), suggesting a shift toward a senescent cell fate. We further analyzed β-galactosidase (β-Gal) activity as a classical marker of senescent cells [67], which confirmed the increased senescence in the ZOL-treated hPdLFs (Figure 3c,d). In addition, increased expression of senescence-associated cytokines such as *IL6*, *IL8* and *COX2* was detected, as well as of the (recently in this context) identified marker *GDF15* (Figure 3e).

Collectively, our data suggest that zoledronic acid induces a relevant stress response by inducing DNA strand breaks, which might contribute to reduced cell survival and promote the cellular senescence of hPdLFs. In line with our results, we used for further investigations 5 µM of zoledronic acid, as this concentration has been experimentally used in other in vitro studies, as well as being close to the clinically applied concentrations [28,68].

### 3.3. Zoledronate Induces a Hyperinflammatory Mechanoresponse but Restrains Osteoclast Activation in Part Modulated by GDF15

A shift to the senescent phenotype may significantly affect the functionality of hPdLFs. This might be particularly relevant in view of their critical role in modulating tissue and bone remodeling during orthodontic tooth movement. Recently, de Sousa et al. [45] demonstrated reduced OTM in ZOL-treated Wistar rats with decreased numbers of activated osteoclasts, while increased necrotic areas were observed in the PdL. Subsequently, we aimed to investigate the influence of ZOL on the mechanoresponse of hPdLFs, more specifically focusing on the induction of pro-inflammatory processes and the activation of OCs.

**Figure 4:**
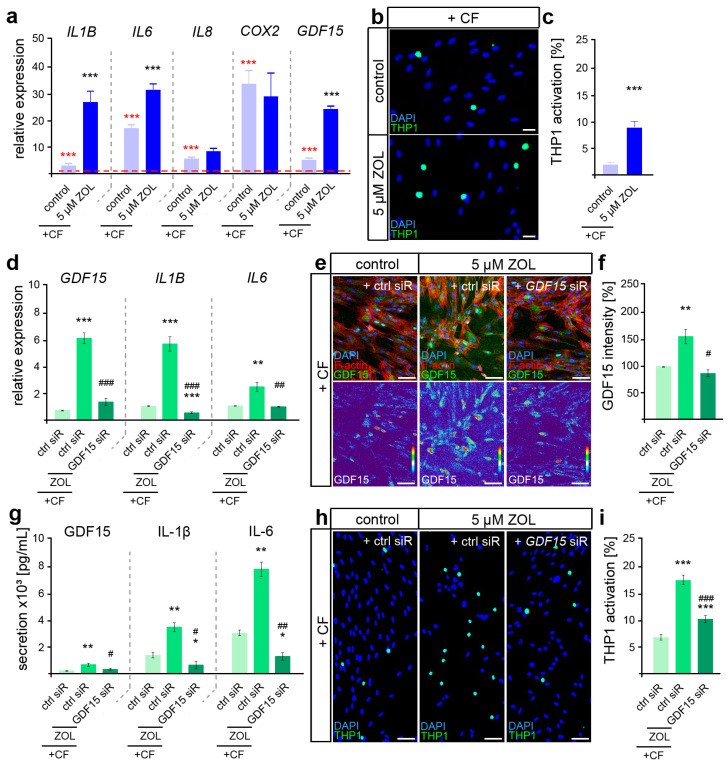
ZOL induced a hyperinflammatory mechanoresponse partially modulated by GDF15. (**a**) Two-day treatment of hPdLFs with 5 µM zoledronic acid (ZOL) led to increased expression of genes encoding pro-inflammatory cytokines in compressed (+CF) hPdLFs. Expression levels are normalized to unstressed controls (red dotted line, red significance indexes). (**b**,**c**) THP1 activation assay revealed increased activation of monocytic THP1 cells (green) by compressed hPdLFs. Nuclei are stained with DAPI (blue). Results are displayed as percentage of THP1 cells per DAPI-positive cells. (**d**) SiRNA-mediated GDF15 knockdown (*GDF15* siR) resulted in decreased expression levels of pro-inflammatory markers in compressed hPdLFs. (**e**,**f**) GDF15 expression was reduced by *GDF15* siRNA-mediated silencing (**e**, green upper panel, intensity staining lower panel), analyzed in (**f**) as relative intensity to the forced control condition. (**g**) Secretion levels of the analyzed pro-inflammatory cytokines are reduced in forced ZOL-treated hPdLFs due to *GDF15* knockdown. (**h**,**i**) THP1 adhesion assay demonstrates reduced activation of monocytic cells by ZOL-treated compressed hPdLFs after GDF15 knockdown. */# *p* < 0.05; **/## *p* < 0.01; ***/### *p* < 0.001; **/*** in relation to control siR +CF in (**a**,**c**) and in relation to ctrl siR +CF conditions in (**d**,**f**,**g**,**i**), ##/### in relation to ZOL- and ctrl-siR-treated +CF conditions. Student’s *t*-test in (**a**,**b**), one-way ANOVA with post hoc test (Tukey’s) in (**d**,**f**,**g**,**i**). Scale bars: 25 µm in (**b**,**h**), 10 µm in (**e**).

To first determine the difference in the inflammatory mechanoresponse profile of ZOL-treated hPdLFs, quantitative expression analysis of the genes encoding force-modulated cytokines was performed after the application of 24 h of compressive force (Figure 4a). 

Increasing expression levels of all the analyzed genes (*IL1B*, *IL6*, *IL8*, *COX2* and *GDF15*) were detected after the application of compressive force (Figure 4a, red significance indexes). While *IL8* and *COX2* showed no ZOL-dependent changes in their force-related increase, we detected significantly enhanced levels of *IL1B*, *IL6* and *GDF15*, suggesting a specific augmented pro-inflammatory profile in relation to ZOL (Figure 4a, black significance indexes). To functionally examine the inflammatory response of mechanically stressed ZOL-exposed fibroblasts, we further investigated the activation of immune cells (Figure 4b,c). When sensing pro-inflammatory signals, non-adherent monocytic THP1 cells differentiate into adherent macrophages [69], which we investigated in co-culture experiments with stimulated hPdLFs. Thus, the number of adherent THP1 cells was significantly increased in compressed ZOL-treated hPdLFs, confirming the hyper-inflammatory mechanoresponse of PdL fibroblasts exposed to zoledronic acid.

Due to the previously described important functions of GDF15 in modulating the pro-inflammatory response of hPdLFs to compressive stimuli [15], we next aimed to investigate its potential role in the hyperinflammatory response of ZOL-exposed hPdLFs to compressive force. To this end, we used siRNA-mediated gene silencing to knock down *GDF15* (*GDF15* siR) after treatment with ZOL, but prior to compressive force application. The expression of GDF15 was reduced by siRNA-mediated knockdown at the RNA (Figure 4d), protein (Figure 4e,f) and secretory levels (Figure 4g). Under *GDF15* deficiency, the ZOL-induced increased expression of *IL1B* and *IL6* was attenuated in the compressed hPdLFs (Figure 4d). This was also evident in the secretion level of those cytokines (Figure 4g). Furthermore, the ZOL-dependent up-regulated activation of immune cells was reduced in the *GDF15*-deficient hPdLFs (Figure 4h,i), specifically indicating the profound pro-inflammatory role of GDF15 in response to ZOL treatment.

**Figure 5:**
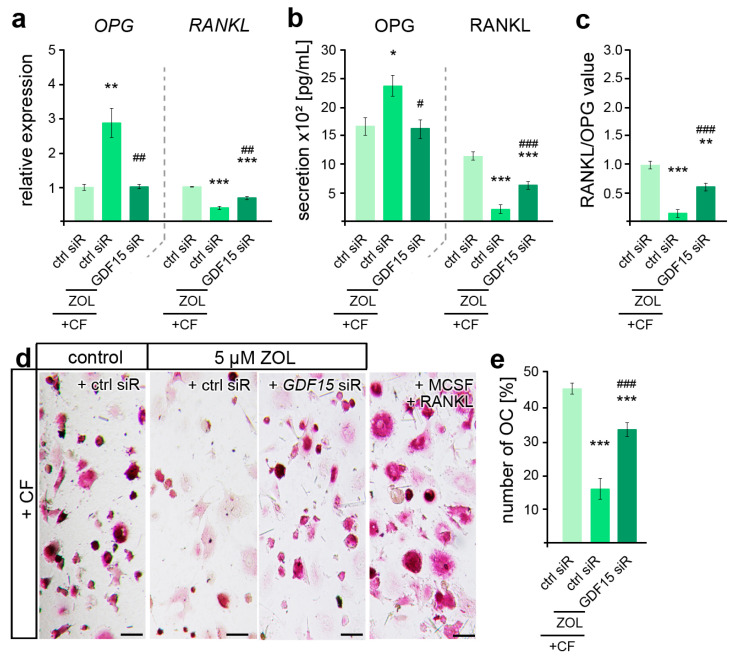
ZOL-induced reduction in osteoclast activation is partially modulated by GDF15. (**a**,**b**) Zoledronic acid (ZOL, 5 µM, 2 d)-induced changes in *OPG* and *RANKL* gene expression (**a**) and secretion (**b**) are partially reversed by *GDF15* deficiency in compressed (+CF) hPdLFs. (**c**) *GDF15* knockdown leads to increased RANKL/OPG values. The data of (**a**,**c**) relate to the forced control siRNA condition. (**d**,**e**) TRAP staining showing reduced osteoclast differentiation by compressed ZOL- and control siRNA (ctrl siR)-treated hPdLFs partially reversed by *GDF15* knockdown. Displayed as number of osteoclasts per image. MCSF and RANKL treatment was used as positive control for osteoclast differentiation. */# *p* < 0.05; **/## *p* < 0.01; ***/### *p* < 0.001; **/*** in relation to ctrl siR +CF conditions, ##/### in relation to ZOL- and ctrl-siR-treated +CF conditions. One-way ANOVA with post hoc test (Tukey’s). Scale bars: 25 µm.

Given the pleiotropic functions of GDF15 in osteoclast activation, which was found impaired in ZOL-treated rodent OTM models [45,70], we subsequently examined the relevance of GDF15 in hPdLF-mediated stress-dependent OC activation. Thus, quantitative analysis of gene expression revealed ZOL-dependent differences in the force-related decrease in *OPG* and increase in *RANKL*, which could be partially prevented by GDF15 silencing (Figure 5a). These findings were also reflected in the secretion levels of both cytokines (Figure 5b), resulting in an increase in RANKL/OPG values with GDF15 deficiency (Figure 5c). However, the RANKL/OPG value was still reduced compared to the compressed controls that were not treated with ZOL. To further functionally validate the osteoclast differentiation, PMC-precultured THP1 macrophages were stimulated for six days with the medium supernatant of the compressed ZOL-treated *GDF15*-deficient hPdLFs in comparison to the corresponding controls (Figure 5d,e). Macrophagic THP1 cells have the ability to differentiate into osteoclasts when they are exposed to stimulating signals such as RANKL and MCSF [71]. Using TRAP staining of the osteoclasts, we determined a reduced number of OCs in the compressed fibroblasts in relation to ZOL treatment. The silencing of GDF15 partially prevented this ZOL-induced inhibition of OC activation.

Taken together, our data suggest that the cellular characteristics altered by ZOL indeed disturb the biomechanical functionality of hPdLFs and that GDF15 plays a crucial role in this process.

## 4. Discussion

The cells in the periodontal ligament, mainly fibroblasts, contribute considerably to alveolar bone remodeling, particularly in orthodontic therapy [1]. However, a variety of pharmaceuticals may elicit functional interference, particularly by inducing DNA damage and thus negatively affecting the bone degradation or attachment processes, respectively [72]. Here, we show that zoledronic acid markedly reduces the viability and proliferative capacity of human PdL fibroblasts. We further observed a marked elevation of DNA damage, driving cellular senescence. Overall, the altered cellular properties of zoledronic-acid-treated PdL fibroblasts subsequently promote hyperinflammatory responses to applied compressive forces with reduced osteoclast activation. In this context, the zoledronic-acid-induced up-regulation of *GDF15* seems to be particularly relevant to the altered responses to mechanical compression.

The impact of zoledronic acid on viability and proliferative capacity is well described for several cell types, specifically for osteoclasts and osteoclast-like cells [70]. Yet, the dose-dependent limitations in the cell survival and proliferative capacity of PdL fibroblasts observed in this study were consistent with the findings of previous studies on this cell type [38,54,55,73]. In contrast to previous studies on hPdLFs and PdL stem cells (PdLSCs) [54,74], we could show that even a concentration of 0.5 µM zoledronic acid applied for two days led to a higher cell death rate and the reduced cell proliferation of the hPdLFs in vitro. The possible reasons for these varying results could be origin-related differences due to the different sources or suppliers of the PdL cells and zoledronate products used [45,55,70], as well as the material of the culture plates [36]. Nevertheless, in all the comparable studies on cells grown in plates with inelastic bottoms, effects on cell survival and proliferation were observed at zoledronate concentrations that are also commonly applied in clinical settings [28].

Zoledronic acid is a potent inducer of reactive oxygen species and DNA damage, which is clinically used to sensitize cancer cells prior to radiotherapy [38,56]. In accordance with other studies [56,62,63,75], we also detected a dose-dependent increase in DNA strand breaks and H2A.X phosphorylation in the ZOL-treated PdL fibroblasts, pointing to enhanced levels of DNA damage. Increased H2A.X phosphorylation levels are also often associated with cellular senescence [66]. In line with this, we further demonstrated a dose-dependent up-regulation of senescence markers including p21^Waf1/Cip1/Sdi1^ and β-galactosidase, as well as an increased expression in senescence-associated secretory phenotype (SASP)-encoding genes, in the ZOL-treated PdL fibroblasts. Correspondingly, enhanced p21^Waf1/Cip1/Sdi1^, β-galactosidase and SASP levels have also been demonstrated in other cell lines due to zoledronic acid exposure [58,76,77]. In general, but also specific to hPdLFs, senescence is associated with reduced proliferation and the loss of cell viability [78], which is also confirmed by our study’s results.

Konstantonis et al. further reported the decreased osteoblastic differentiation of senescent hPdLFs according to a lower number of calcium deposits being stained using alizarin red [78]. In contrast to the subculture- or irradiation-induced senescence investigated in their study, the zoledronate-related senescence reported here did not resulted in any changes in calcium deposit labeling or the expression of the osteogenic markers *ALPL* and *RUNX2* in the hPdLFs. This is in line with comparable studies on the human osteoblast-like cell lines MG-63 and G-292, which also demonstrated no influence of zoledronic acid on their osteogenic differentiation [79].

Furthermore, it was recently reported that in vitro aged human lung fibroblasts tend to undergo senolysis [39], a highly specific compound-induced death of senescent cells [80], when stimulated with zoledronate. Since we did not specifically analyze the mitotic state of the hPdLFs identified as dead, we can only speculate whether zoledronic acid also induces senolytic effects in PdL fibroblasts subsequent to the induction of senescence. However, progressive senescence compromises cellular functionality, particularly due to the increased pro-inflammatory senescence-associated secretion profile [39]. Here, we detected an increased expression of SASP-encoding genes in the ZOL-treated hPdLFs, in particular *IL6*, *IL8* and *COX2*. We observed similar findings for *COX2*, but not for *IL6*, in a previous study when ZOL exposure was performed in a starvation medium [68]. Comparable in vitro studies on fibroblasts, osteoblast- and osteocyte-like cells also revealed increasing pro-inflammatory states due to zoledronic acid treatment [57,68,79]. In vivo, the prolonged application of zoledronate specifically enhanced pro-inflammatory cytokines such as IL-1β and tumor necrosis factor α (TNF-α) in the rat periodontium [59]. In this study, we now also demonstrate a significant increase in GDF15 levels after treatment with zoledronate.

Patel et al. investigated the bone turnover in bisphosphonate-treated multiple myeloma patients in remission, showing decreased serum levels of GDF15 six months after a single dose of zoledronic acid [81]. However, a direct effect like in our study cannot be assumed. More recently, GDF15 has been proposed to be an important senescence-associated secretory phenotype and aging marker [82]. Interestingly, an eight-week zoledronate treatment of aged mice significantly lowered the SASP, including the expression level of *Gdf15*, indicating also an adverse effect. However, oxidative stress, a well-known source of DNA damage, induced by radiation highly increased the GDF15 levels in oral squamous cell carcinoma cells [83]. Thus, the impact of zoledronate on GDF15 potentially highly depends on the initial state of the tissue or cells besides exposure time and compound concentration.

Given their important role in modulating the inflammatory mechanoresponse to compressive stimuli that typically occurs during orthodontic treatment [1,8], a pro-inflammatory shift in the basic cellular profile might be of significant relevance to periodontal fibroblasts. We recently demonstrated an increased pro-inflammatory response of ZOL-treated hPdLFs to short but excessive compressive stress in an in vitro periodontal disease model [68]. In this study, the potential effects of ZOL on shortly compressed PdL fibroblasts were only shown when they were co-stimulated with IL-1β. Here, we could also detect the increasing effect of ZOL on mechano-modulated PdL inflammation when applying 24 h compressive force, even without an additional inflammatory stimulus. Compared with 3 h [68], 24 h of mechanical compression generally results in a more profound pro-inflammatory response and an up-regulation of IL-1β [84].

The increased expression and secretion levels of IL-1β and IL-6 in 24 h compressed hPdLFs due to ZOL treatment are in line with studies in other cells [85,86]. However, their increase seemed to be at least partially dependent on GDF15, as siRNA-mediated GDF15 knockdown resulted in decreased levels of both inflammatory markers. This correlates with the results of our recent study showing even without ZOL treatment the significant influence of GDF15 on *IL1B* and *IL6* expression, as well as the activation of THP1 monocytic cells in compressed hPdLFs [15]. However, since we observed increased intracellular as well as extracellular GDF15 levels due to ZOL exposure, we can only speculate about the effects and the exact mechanism.

Nevertheless, zoledronic acid and compressive force seems to impact similar pro-inflammatory key players in hPdLFs, and GDF15 might be an important inflammatory modulator in the complex interplay of both extrinsic stressors. The signaling pathways potentially affected could include the intracellular mediators of the SMAD family, which are activated by the phosphorylation processes via TGF-β/BMP receptors and subsequently transmit appropriate signals to the nucleus [87]. In this regard, a potential stress-signaling connection between both extrinsic influences already seems very likely for the TGF-β family in other cellular contexts [88]. We recently identified ALK1/2/5 as potential GDF15 receptors in hPdLFs [16], which are well described as acting in the SMAD signaling pathway. However, despite the functions of the mature secreted form of GDF15, intracellular activities of the immature GDF15 protein are also described [89].

The excessive immune response of PdLFs could impair bone remodeling due to the complex interaction of further immune cells, osteoblasts and osteocytes in the activation of bone-resorbing osteoclasts [8,49,51,52]. In particular, the RANKL/OPG level in the surroundings seems to be of great importance to their differentiation into mature osteoclasts. We have recently demonstrated that ZOL affects the mechanical response of hPdLFs to tensile forces by influencing the RANKL/OPG levels toward increased OC activation, which is rather detrimental since bone formation is normally promoted by this force [36]. Compressive force promotes bone resorption, but in this study, we could show that ZOL appears to have a disadvantageous effect on OC maturation by decreasing the RANKL/OPG ratio. This seems to be consistent with the general mechanism of ZOL, whose characteristic is reduced OC activation [31]. In this context, our study highlights an additional mechanism via the PdLFs with GDF15 a potential modulator, since siRNA-mediated down-regulation partially balanced the ZOL-induced reduction in the OC activation of the compressed hPdLFs. Interestingly, our results are in conflict with the study by Li et al. [17], who reported that GDF15 seemed to be relevant to the force-induced elevated RANKL/OPG values. In addition to potential influences such as the origin of the cell material and differences in the experimental design, we speculate that zoledronic acid influences a variety of other regulators in addition to GDF15 during the mechanical stress response, and that this in turn influences GDF15-modulated processes.

However, our study is limited in regard to the in vitro design and the specific experimental conditions. Although we used a zoledronic acid concentration typically achieved in the tissue when using ZOL infusion in clinical settings [28,68], this may insufficiently mimic the in vivo effect. Furthermore, we focused on 24 h-compressed hPdLFs given the relatively robust inflammatory response and high GDF15 expression. Future studies could focus on correlating these findings with the processes in vivo and further address the role of the intracellular and extracellular GDF15 protein states.

Overall, this study provides an insight into how human periodontal cells respond to compression stimuli under zoledronate treatment and highlights the important influence of GDF15 in this context. Due to the increasing interest in GDF15-regulating drugs in a variety of diseases, the factor also could become relevant in the future to the patient-specific treatment of orthodontic patients under zoledronic acid. After a subsequent in vivo validation of our results and the potential effects of GDF15, a pre-therapeutic analysis of this factor could be used to perform an additional risk assessment in the context of orthodontic therapy. In addition, the possible role of GDF15 with regard to antiresorptive-drug-induced osteonecrosis of the jaw as the most critical intraoral side effect of ZOL therapy could be of future interest.

## 5. Conclusions

Zoledronate can inhibit tooth movement by directly inhibiting bone-resorbing cells and, as our study has now shown, by indirectly affecting their activation by influencing the cellular properties and mechanobiological functions of the periodontal ligament fibroblasts. Here, we demonstrated that zoledronic acid specifically triggers DNA damage and cellular senescence, potentially triggering the hyperinflammatory mechanoresponse with reduced osteoclast activation. However, this could trigger possible negative side effects in the orthodontic treatment of bisphosphonate patients. With GDF15 as a potential modulator that promotes these ZOL-related changes, we have identified a possible target for future patient-specific therapeutic strategies.

## Figures and Tables

**Table 1:** Forward and reverse qPCR primer sequences of human genes indicated in 5′-3′ direction.

Gene	Gene Symbol	NCBI Gene ID	Primer Sequence
Alkaline phosphatase	*ALPL*	249	ACTGCAGACATTCTCAAA GAGTGAGTGAGTGAGCA
C-X-C motif chemokine ligand 8	*IL8*	3576	TTGGCAGCCTTCCTGATTTCT GGTCCACTCTCAATCATCTCA
Growth differentiation factor 15	*GDF15*	3576	CCGAAGACTCCAGATTCCGA CCCGAGAGATACGCAGGTG
Interleukin 1 beta	*IL1B*	3553	CGAATCTCCGACCACCACTA AGCCTCGTTATCCCATGTGT
Interleukin 6	*IL6*	3569	CATCCTCGACGGCATCTCAG TCACCAGGCAAGTCTCCTCA
Prostaglandinendoperoxide synthase 2	PTGS2 (alias *COX2*)	4743	GATGATTGCCCGACTCCCTT GGCCCTCGCTTATGATCTGT
RUNX family transcription factor 2	*RUNX2*	6146	CCCACGAATGCACTATCC GGACATACCGAGGGACA
TNF receptor superfamily member 11b	TNFRSF11B (alias *OPG*)	4982	GAAGGGCGCTACCTTGA GCAAACTGTATTTCGCTC
TNF superfamily member 11	TNFSF11 (alias *RANKL*)	8600	ATCACAGCACATCAGACAGA TCATTTATGGAACAGATGGG

## Data Availability

The datasets from this study are available upon reasonable request from the corresponding author.
